# Innate RIG-I signaling restores antigen presentation in tumors and overcomes T cell resistance

**DOI:** 10.15698/cst2021.02.242

**Published:** 2021-01-18

**Authors:** Beatrice Thier, Annette Paschen

**Affiliations:** 1 Department of Dermatology, University Hospital Essen, University of Duisburg-Essen, Essen, Germany.; 2 German Cancer Consortium (DKTK), University Hospital Essen, Essen, Germany.

**Keywords:** tumor, HLA class I, T cell resistance, RIG-I, interferon, immune checkpoint blockade

## Abstract

In recent years, therapy with immune modulating antibodies, termed immune checkpoint blockade (ICB), has revolutionized the treatment of advanced metastatic melanoma, yielding long-lasting clinical responses in a subgroup of patients. But despite this remarkable progress, resistance to therapy represents a major clinical challenge. ICB efficacy is critically dependent on cytotoxic CD8+ T cells targeting tumor cells in an HLA class I (HLA-I) antigen-dependent manner. Transcriptional suppression of the HLA-I antigen processing and presentation machinery (HLA-I APM) in melanoma cells leads to HLA-I-low/-negative tumor cell phenotypes escaping CD8+ T cell recognition and contributing to ICB resistance. In general, HLA-I-low/-negative tumor cells can be re-sensitized to T cells by interferons (IFN), augmenting HLA-I APM expression. However, this mechanism fails when melanoma cells acquire resistance to IFN, which recently turned out as a key resistance mechanism in ICB, besides HLA-I APM suppression. Seeking for a strategy to overcome these barriers, we identified a novel mechanism that restores HLA-I antigen presentation in tumor cells independent of IFN (Such *et al.* (2020) J Clin Invest, doi: 10.1172/JCI131572). We demonstrated that tumor cell-intrinsic activation of the cytosolic innate immunoreceptor RIG-I by its synthetic ligand 3pRNA overcomes transcriptional HLA-I APM suppression in patient-derived IFN-resistant melanoma cells. *De novo* HLA-I APM expression is IRF1/IRF3-dependent and re-sensitizes melanoma cells to autologous cytotoxic CD8+ T cells. Notably, synthetic RIG-I ligands and ICB synergize in T cell activation, suggesting combinational therapy could be an efficient strategy to improve patient outcomes in melanoma.

## HLA CLASS I-LOW/-NEGATIVE MELANOMA CELL PHENOTYPES AND DEFECTIVE INTERFERON SIGNALING IN RESISTANCE TO IMMUNE CHECKPOINT BLOCKADE

Melanoma immunotherapy exploits the capability of cytotoxic CD8+ T cells to selectively kill tumor cells. This selectivity is achieved by the T cell receptor binding to specific HLA class I (HLA-I) antigen complexes on melanoma cells. However, in the tumor microenvironment T cell activity is blocked by the inhibitory co-receptor PD-1 (immune checkpoint), signaling upon engagement of its ligand PD-L1 on melanoma cells. Therapeutic antibodies have been developed that disturb the inhibitory PD-1/PD-L1 axis and release T cells from suppression. Remarkably, antibody-based therapy, termed immune checkpoint blockade (ICB), induces clinical responses in 40-50% of melanoma patients with advanced metastatic disease. But despite this tremendous clinical progress, still the majority of patients does not respond at all to ICB (primary resistance) or relapse after initial therapy response (acquired resistance).

**Figure 1:**
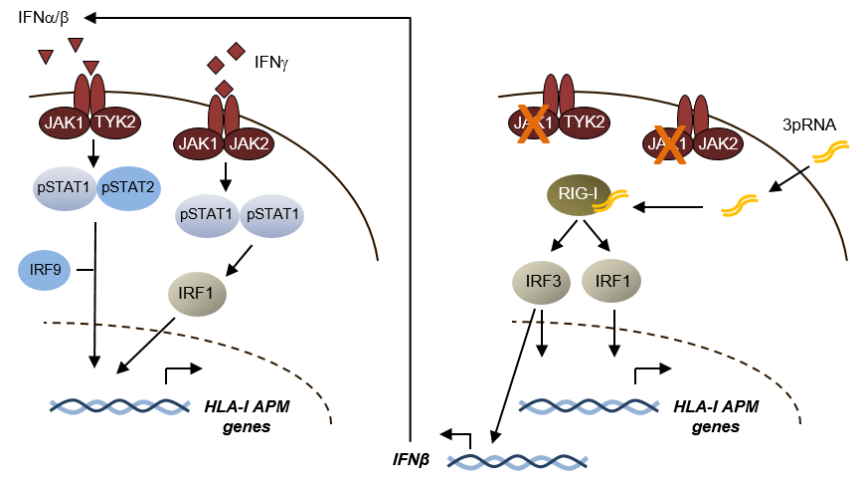
FIGURE 1: IFN-dependent and –independent control of HLA-I antigen processing and presentation genes. Schematic showing signaling pathways and transcriptional regulators involved in the control of HLA-I APM expression. Left, Type I/II interferon signaling; right, RIG-I signaling.

We aimed to elucidate resistance mechanisms in ICB in order to improve patient outcome and identified the development of poorly immunogenic HLA-I-low and HLA-I-negative tumor phenotypes as a barrier to effective immunotherapy. Transcriptional suppression of the HLA-I antigen processing and presentation machinery (HLA-I APM), including *HLA-A, HLA-B, HLA-C, B2M, TAP1, TAP2, TABP, LMP7* and *LMP9* genes, gives rise to those phenotypes that escape recognition by cytotoxic CD8+ T cells. Analyzing distinct melanoma transcriptomic data sets and annotated clinical data, we found low HLA-I APM expression levels associated with ICB resistance. Type I (IFNα/β) and type II (IFNγ) interferons (IFN) are well defined for their capability to counteract HLA-I downregulation by activation of JAK/STAT signaling pathways (**Figure 1**). However, mutational inactivation of JAK1, a kinase involved in IFN-I/-II signaling, enables melanoma cells to preserve their immune-evasive HLA-I-low/-negative phenotype in an IFN-rich microenvironment. Defective IFN signaling was recently defined as a key resistance mechanism in ICB which led us to seek for strategies enhancing HLA-I antigen processing and presentation by IFN-dependent and IFN-independent mechanisms.

## TARGETING RIG-I TO RESTORE TUMOR ANTIGEN PRESENTATION AND OVERCOME T CELL RESISTANCE INDEPENDENT OF INTERFERONS

We speculated that innate pattern recognition receptors, controlling responses to viral infections, could be a mechanistic tool to efficiently induce HLA-I APM expression. As such, the cytosolic RNA helicase RIG-I responds to short 5′-triphosphorylated double-stranded viral RNA and triggers expression of a gene set involved in detection and elimination of virus-infected cells. In fact, in distinct patient models we observed that activation of RIG-I by its ligand 3pRNA, a synthetic viral RNA mimetic, strongly enhanced HLA-I APM expression in melanoma cells and increased their sensitivity towards autologous CD8+ T cells. RIG-I activation also triggered the expression of IFNβ, suggesting that autocrine and paracrine IFN-I signaling in melanoma cells elicited upregulation of the distinct HLA-I APM components. Strikingly, we detected similar effects in IFN-I-sensitive and IFN-I-resistant tumor cells. 3pRNA treatment even forced *de novo* HLA-I APM expression in patient-derived JAK1 mutant melanoma cells and restored their T cell sensitivity, demonstrating that activated RIG-I triggered an IFN-independent salvage pathway capable of overcoming tumor cell-intrinsic T cell resistance. Mechanistic studies revealed that this process was dependent on the transcriptional activators IRF1 and IRF3 (**Figure 1**). In addition to its effects on antigen presentation, RIG-I activation also induced *de novo* expression of several T cell-attracting chemokines in IFN-sensitive and -resistant melanoma cells. Pronounced effects on tumor antigen presentation and T cell attraction were observed also upon intratumoral 3pRNA application in different xenotransplant melanoma models. Consistent with our *in vitro* and *in vivo* studies we found expression of HLA-I APM genes, chemokines, and markers of T cell activation enhanced in *RIG-I (DDX58)*-high tumors of the TCGA melanoma cohort. A strong correlation between expression of *RIG-I (DDX58)* pathway genes (*DDX58, IRF1, IRF3*) and HLA-I APM genes, was also detected in melanoma biopsies from ICB-treated patients. This prompted us to study the combination of 3pRNA and immune checkpoint blocking antibodies for its capacity to synergize in T cell activation. To this end, we took advantage of an autologous melanoma model from a patient with primary ICB resistance, consisting of CD8+ tumor infiltrating lymphocytes (TILs) showing positivity for PD-1 and TIGIT, another inhibitory co-receptor, and melanoma cells expressing the corresponding receptor ligands. In this model we demonstrated that HLA-I APM upregulation by 3pRNA and anti-PD-1/anti-TIGIT antibodies synergistically enhanced the reactivity of CD8+ TILs towards autologous melanoma cells.

Overall, our study linked transcriptional HLA-I APM suppression in melanoma to ICB resistance and nominated RIG-I as a druggable therapeutic target to restore antigen processing and presentation and overcome T cell resistance of IFN-sensitive and IFN-resistant tumor cells. The observed synergistic effects of combined RIG-I activation and ICB in T cell activation provide a strong rational for ongoing clinical trials combining intratumoral injection of synthetic RIG-I ligands with anti-PD-1 or anti-PD-L1 antibodies. So far, this application is limited to accessible melanoma skin or lymph node lesions, indicating that development of specific transport systems allowing for tumor-specific drug delivery to distant metastases is urgently needed. Researchers should be encouraged to develop such carrier systems by the fact that, HLA-I APM-low phenotypes are involved not only in resistance to ICB but also other types of immunotherapy and can be detected also in other tumor entities besides melanoma.

